# Anxiety Online—A Virtual Clinic: Preliminary Outcomes Following Completion of Five Fully Automated Treatment Programs for Anxiety Disorders and Symptoms

**DOI:** 10.2196/jmir.1918

**Published:** 2011-11-04

**Authors:** Britt Klein, Denny Meyer, David William Austin, Michael Kyrios

**Affiliations:** ^1^National eTherapy CentreFaculty of Life and Social SciencesSwinburne UniversityMelbourneAustralia; ^2^Brain and Psychological Sciences Research CentreFaculty of Life and Social SciencesSwinburne UniversityMelbourneAustralia; ^3^eTherapy Research UnitFaculty of Life and Social SciencesSwinburne UniversityMelbourneAustralia

**Keywords:** eTherapy, Internet interventions, e-mental health, cognitive behavior therapy, generalized anxiety disorder, panic disorder, obsessive compulsive disorder, social anxiety disorder, posttraumatic stress disorder, self-help, fully automated, Web treatment

## Abstract

**Background:**

The development of e-mental health interventions to treat or prevent mental illness and to enhance wellbeing has risen rapidly over the past decade. This development assists the public in sidestepping some of the obstacles that are often encountered when trying to access traditional face-to-face mental health care services.

**Objective:**

The objective of our study was to investigate the posttreatment effectiveness of five fully automated self-help cognitive behavior e-therapy programs for generalized anxiety disorder (GAD), panic disorder with or without agoraphobia (PD/A), obsessive–compulsive disorder (OCD), posttraumatic stress disorder (PTSD), and social anxiety disorder (SAD) offered to the international public via Anxiety Online, an open-access full-service virtual psychology clinic for anxiety disorders.

**Methods:**

We used a naturalistic participant choice, quasi-experimental design to evaluate each of the five Anxiety Online fully automated self-help e-therapy programs. Participants were required to have at least subclinical levels of one of the anxiety disorders to be offered the associated disorder-specific fully automated self-help e-therapy program. These programs are offered free of charge via Anxiety Online.

**Results:**

A total of 225 people self-selected one of the five e-therapy programs (GAD, n = 88; SAD, n = 50; PD/A, n = 40; PTSD, n = 30; OCD, n = 17) and completed their 12-week posttreatment assessment. Significant improvements were found on 21/25 measures across the five fully automated self-help programs. At postassessment we observed significant reductions on all five anxiety disorder clinical disorder severity ratings (Cohen d range 0.72–1.22), increased confidence in managing one’s own mental health care (Cohen d range 0.70–1.17), and decreases in the total number of clinical diagnoses (except for the PD/A program, where a positive trend was found) (Cohen d range 0.45–1.08). In addition, we found significant improvements in quality of life for the GAD, OCD, PTSD, and SAD e-therapy programs (Cohen d range 0.11–0.96) and significant reductions relating to general psychological distress levels for the GAD, PD/A, and PTSD e-therapy programs (Cohen d range 0.23–1.16). Overall, treatment satisfaction was good across all five e-therapy programs, and posttreatment assessment completers reported using their e-therapy program an average of 395.60 (SD 272.2) minutes over the 12-week treatment period.

**Conclusions:**

Overall, all five fully automated self-help e-therapy programs appear to be delivering promising high-quality outcomes; however, the results require replication.

**Trial Registration:**

Australian and New Zealand Clinical Trials Registry ACTRN121611000704998; http://www.anzctr.org.au/trial_view.aspx?ID=336143 (Archived by WebCite at http://www.webcitation.org/618r3wvOG)

## Introduction

Anxiety disorder is a generic term given to a group of specific disorders that are typically characterized by fear, worry, and phobic responses. The main anxiety disorder types are generalized anxiety disorder (GAD), panic disorder with or without agoraphobia (PD/A), obsessive–compulsive disorder (OCD), posttraumatic stress disorder (PTSD), and social anxiety disorder (SAD). These disorders are highly prevalent mental health conditions that have deleterious effects on a person’s life, including substantial personal, social, and occupational impairment, and are often associated with considerable comorbidity [1-4] resulting in significant economic costs for the individual and society. In the Australian National Mental Health Survey [2], only around one-third of those with an anxiety disorder (37.8%) reported making use of services over the previous 12 months for their mental health problems [5].

Cognitive behavior therapy (CBT) has been shown to be an effective treatment for GAD, PD/A, OCD, PSTD, and SAD. Face-to-face CBT for these anxiety disorders typically involves 60 to 90 minutes of treatment per week over 9–12 weeks, including psychoeducation, anxiety management (eg, relaxation techniques), cognitive and exposure therapy, and relapse prevention [6]. Nevertheless, this form of specialized treatment is unavailable to many of those affected due to a shortage of suitably qualified health care professionals (especially in regional and rural areas), fee-for-service costs, and the stigma attached to seeing a mental health professional [7,8].

The development of e-therapy or e-mental health interventions (delivery of mental health interventions and services via information and communication technologies) has grown exponentially over the past decade, and is one way of delivering CBT that overcomes the commonly cited obstacles to treatment provision [9]. There are now hundreds of e-mental health interventions designed to treat or prevent mental illness and to enhance well-being. A helpful practitioner and consumer resource that provides information and quality ratings for over 180 e-physical health and e-mental health interventions can be accessed via Beacon [10], an online portal to eHealth interventions [11].

The most common type of e-mental health intervention is the Internet- or Web-based intervention or e-therapy (see [12]). e-Therapy programs can be broadly categorized as being self-help or therapist-assisted, and hundreds have been evaluated across a range of mental health disorders and symptoms, including panic disorder (eg, [13,14], SAD (eg, [15]), PTSD and symptoms (eg, [8,16,17]), anxiety prevention (eg, [18,19]), depression and depressive symptoms (eg, [20-24]), insomnia (eg, [25]), and alcohol issues (eg, [26]). Additionally, therapist-assisted e-therapy treatment programs have been found to be as effective as best-practice face-to-face therapy [13,27]. Numerous reviews [28-30] and meta-analyses (eg, [31,32]) attest to the general effectiveness of e-therapies based on validated therapeutic models such as CBT.

Although hundreds of e-therapy programs have been developed, the vast majority are generally accessible only via participation in research trials. In addition, most of the programs developed are singular offerings rather than broad-based virtual clinics offering multiple services. However, several groups offer an array of e-therapy programs contained within a single platform, such as e-hub [33], eCentreClinic [34], and Anxiety Online [35]. e-hub, operating through the Australian National University, Canberra, Australia, provides a variety of open-access self-help programs for mental health and well-being, such as MoodGYM, BluePages, BlueBoard, and e-couch [36], to the worldwide public. However, these e-mental health programs were largely designed to prevent ill health, rather than to treat clinical populations, and online therapist assistance is not offered (although BlueBoard, an Internet support group facility, includes human moderators who oversee consumer postings and appropriate online behaviors). On the other hand, the eCentreClinic, operating through Macquarie University, Sydney, Australia, offers a range of self-help and therapist-assisted e-therapy treatment programs for the anxiety disorders and depression. However, access is restricted to participation in research trials, opened only to the Australian public and at different times during the year.

Anxiety Online, operating through the National eTherapy Centre at Swinburne University of Technology, and funded by the Australian Government Department of Health and Ageing, provides to the international public a full-service, open-access, virtual psychology clinic for anxiety disorders. More specifically, Anxiety Online comprises four major components: (1) an open-access psychoeducational website that provides information about Anxiety Online, anxiety disorders (symptoms, prevalence, how and where they are treated), links to useful resources, and an entry/registration point for consumers, health care practitioners, and administrators, (2) a freely available online psychological assessment and referral system (e-PASS) that assesses the person for symptoms associated with 21 disorders in the Diagnostic and Statistical Manual of Mental Disorders: DSM-IV-TR, 4th edition, text revision (DSM-IV-TR) [37] (ie, PD/A, agoraphobia without history of panic disorder, SAD, specific phobia, GAD, PTSD, OCD, depression, anorexia nervosa, bulimia nervosa, binge eating disorder, somatization, body dysmorphic disorder, pathological gambling, insomnia, hypersomnia, alcohol dependence, and substance dependence—cannabis, opioids, sedatives, and stimulants), (3) five interactive, fully automated, 12-module self-help or therapist-assisted (via email) e-therapy treatment programs for GAD, PD/A, PTSD, OCD, and SAD, and (4) online e-therapist/CBT training programs and a health care practitioner portal.

Before commencing work as an e-therapist at the National eTherapy Centre, all prospective therapists are provided online e-therapy and CBT training and must pass a competency-based assessment (see [38] for more details). Many of the Anxiety Online e-therapists are postgraduate psychology students from various Australian universities who are undertaking an e-therapy psychological internship or placement. At a minimum, all have provisional registration as a psychologist. Anxiety Online also provides health care professionals worldwide (eg, general practitioners, psychologists, social workers, mental health nurses, aboriginal health workers, and psychiatrists) free access to the Anxiety Online programs (visit [39] to register).

Anxiety Online was launched for the international public in October 2009. This paper reports on the pre- to posttreatment outcome results for the completers of the five fully automated self-help treatment programs from October 2009 to April 2011. The goal of the Anxiety Online service is to increase access to mental health services by reducing the common obstacles and to provide consumers with choice in regard to treatment, as is the case for real-world settings. Therefore, we used a naturalistic design to evaluate the mental health treatment outcomes. The primary treatment outcome measure was the anxiety disorder severity ratings, with secondary outcome measures relating to general psychological distress levels, total number of DSM-IV-TR [37] diagnoses, confidence in managing one’s own mental health care, and quality of life. We expected that after completing one of the five Anxiety Online treatment programs, participants would show decreases in their anxiety disorder severity rating, general psychological distress levels, and the total number of mental health diagnoses at posttreatment, as well as improvements in confidence in managing their own mental health care and quality of life.

## Methods

### Participants and Flow

Anxiety Online is an open-access website platform. We recruit participants via periodic Facebook advertisements, referral links on other mental health websites, use of local and national media, and presentations and brochure mail-outs to health care practitioners and consumer groups.

When visiting Anxiety Online, participants wanting to undertake e-PASS are first required to register and consent to the Anxiety Online terms and conditions [40]. After providing consent, participants are taken to e-PASS, which is the gateway to the fully automated self-help and therapist-assisted treatment programs. e-PASS was designed to ensure that all participants were offered an appropriate e-therapy treatment program based on their reported symptoms, as well as a way to help them identify whether they are experiencing difficulties within a range of psychological symptoms and disorders. In addition to addressing 21 DSM-IV-TR [37] disorders, a variety of demographic and personal information (eg, whether they are currently accessing mental health treatment) is recorded.

The key inclusion criteria for access to the treatment programs are being 18 years of age or older, completing e-PASS, and having either a subclinical or clinical diagnosis of at least one of GAD, PD/A, OCD, PTSD, or SAD. From October 2009 until April 2011, there were 7140 legitimate e-PASS pretreatment completions. The Anxiety Online data file initially contained 7245 completed pretreatment e-PASS administrations; however, 105 were removed (ie, 81 reported being under 18 years of age; and 24 were identified as health care professionals or researchers not interested in using Anxiety Online for their own personal purposes). From the 7140 e-PASS pretreatment completers, 168 did not have any disorder or symptoms and an additional 593 did not have a clinical or subclinical diagnosis of GAD, PD/A, OCD, PTSD, or SAD, for which Anxiety Online has treatment programs. This left 6379 consumers being offered an Anxiety Online treatment program. From this, 2660 elected to start a program and 3719 elected not to. The overall Anxiety Online e-therapy program acceptance rate was therefore 42% (2660/6379). It is important to note, however, that only 2986 of 6379 participants had an anxiety disorder as their primary diagnosis and, therefore, 89% (2660/2986) of participants with a primary anxiety disorder elected to commence one of the e-therapy programs. This is important because e-PASS strongly encourages participants, via their e-PASS feedback report, to seek treatment for their primary condition first.

Of the 2660 who started an e-therapy program, 75 elected to take the therapist-assisted version (due to the small numbers, these data are not presented in this paper). Of the remaining 2585, at time of data analyses 350 of the participants in the fully automated self-help program were still in progress; thus, the total number of participants who had completed their 12-week treatment period was 2235. From this sample, 832 commenced GAD Online, 406 commenced Panic Stop!, 168 commenced OCD Stop!, 227 commenced PSTD Online, and 602 commenced SAD Online. Anxiety Online also collects e-PASS data from registered program users every year for 5 years, and this follow-up data will be reported in due course. The procedures for reporting of the Anxiety Online data were approved by the Swinburne University Human Research Ethics Committee. Trial registration was obtained retrospectively because Anxiety Online is an ongoing open-access mental health service rather than a pure research trial. The program automatically collects consumer data regarding treatment outcomes via e-PASS and therefore differs from the traditional trial study design that has a clear start and end date.

### Measures

Assessment included an online automated self-report clinical interview (e-PASS) assessing for 21 DSM-IV-TR [37] disorders, plus several other online questionnaires at pre- or posttreatment, or both.

#### Online Psychological Assessment and Referral System

e-PASS (B Klein, DPsych (Clinical), unpublished data, September 2010) is an online self-report diagnostic tool that assesses for 21 DSM-IV-TR [37] disorders and serves as the gateway into the fully automated self-help and therapist-assisted treatment programs. Although e-PASS can be completed over several sittings, it must be completed within a 24-hour period. e-PASS is automated and consists of over 540 items directly using the criteria specified in the DSM-IV-TR [37]. In addition to addressing 21 DSM-IV-TR [37] disorders, a variety of demographic (and personal) questions are asked, as well as several screening items (eg, suicide, distorted thinking). It also checks for whether medical conditions and substance affects may better account for reported symptoms. For those who report suicidal ideation or distorted thinking patterns, e-PASS strongly recommends that the test taker cease completing e-PASS and contact a more appropriate service (referral sources are provided).

e-PASS is a complicated system, using both a categorical and dimensional approach to diagnosis, as well as branching logic and algorithmic scoring rules to minimize the number of irrelevant items presented, and is sensitive to other possible causes for symptoms being reported (eg, medical conditions). As a result, the number and types of items presented differ depending on the symptoms being endorsed and this, in turn, affects the feedback provided to the participant via the comprehensive personalized report generated on completion of e-PASS.

The e-PASS feedback report includes likely primary diagnosis, any likely secondary diagnoses (ranked according to number of symptoms and self-reported severity), and whether each likely diagnosis is within a clinical or subclinical range. Disorders in the subclinical range refer to those individuals who report most, but not all, of the required DSM-IV-TR criteria or, alternatively, report all of the necessary DSM-IV-TR criteria but provide low distress and interference ratings regarding their specific disorder symptoms. People with subclinical disorders are symptomatic (or subthreshold) but do not meet full DSM-IV-TR criteria for a clinical disorder. Disorders at the clinical level are further defined as mild, moderate, or severe, and participants are given hyperlinked or pop-up information explaining in plain language what each of these terms means. Likely clinical disorder severity ratings range from 0 (absence of any symptoms) to 8 (very severe clinical disorder) and constitute one of the main outcome variables for this study. Likely clinical disorder severity scores below 3.50 are given a subclinical label and rating. Feedback reports strongly recommend that consumers address their primary condition first, but ultimately what course of action or treatment participants undertake remains their choice (ie, Anxiety Online enables access to treatment programs for each consumer’s primary diagnosis and any secondary diagnoses). Participants are also provided with a summary of the symptoms for each condition they have, and qualifiers are provided where appropriate (eg, chronicity of PTSD). Consumers are also provided a recommended course of action and multiple referral options. As individuals remain completely free to choose whatever course of action they desire, within the confines of the symptoms reported, Anxiety Online is a participant choice-based system. Nevertheless, it also provides the participant with detailed and evidence-based guidance and recommendations.

e-PASS is undergoing psychometric validation and qualitative evaluation, and the pilot and preliminary data suggest it is an acceptable and valid diagnostic tool (B Klein, DPsych (Clinical), unpublished data, September 2010, [41]), although caution is still warranted until the full and detailed study is published. Basic community-based validation results also attest to its validity. Using the results from the current study, at posttreatment 64 participants reported that they sought confirmation of their e-PASS diagnoses with an external source (n = 33 with a psychologist, n = 16 with a medical doctor, n = 6 with a website, n = 4 with a counselor, n = 4 with a friend, and n = 1 with a book). The overall agreement rate was 95% (61/64). The three sources where agreement was not reached were a medical doctor (n = 1), a website (n = 1), and a friend (n = 1).

#### Online Questions/Questionnaire: Self-Report

*Kessler-6* (K6) [42] is a brief 6-item self-report measure, using a Likert-type scale from 1 to 5, measuring nonspecific psychological distress over the last 30 days [42]. Scores range from 6 to 30, with higher scores indicating greater psychological distress. Normative data (G Andrews, MD, written communication, August 2010) suggest that scores between 6 and 11 indicate low distress levels (71.7% of the population); scores between 12 and 15 indicate moderate distress levels (16.6% of the population); scores between 16 and 19 indicate high distress levels (7.16% of the population); and scores between 20 and 30 indicate very high distress levels (2.5% of the population). The K6 has demonstrated strong psychometric properties (eg, [42,43])

*Number of e-PASS diagnoses* is the total number of e-PASS diagnoses (at clinical and subclinical levels) as assessed by e-PASS at pre- and posttreatment assessment.

*Confidence in managing mental health* is a single-question self-report item asking participants to rate their overall level of self-confidence in managing their own mental health. Scores are anchored (1 = very poor, 2 = poor, 3 = neither poor nor good, 4 = good, 5 = very good), with higher scores indicating greater self-confidence.

*Quality of life* is a single-item self-report question asking participants to rate their overall quality of life. Scores are anchored (1 = very poor, 2 = poor, 3 = neither poor nor good, 4 = good, 5 = very good), with higher scores indicating a higher quality of life.

The two *e-Therapy treatment satisfaction questions* ask participants to rate (1) how satisfied they were with the e-therapy program, using a scale from 1 (not at all) to 5 (very highly), and (2) how much they liked the e-therapy treatment program, using a scale of 0 (not at all), 2 (a little), 4 (somewhat), 6 (quite a lot), and 8 (very much so) at posttreatment.

### Design

The five fully automated self-help e-therapy treatment programs were trialed using a pre- to posttreatment quasi-experimental (participant choice) naturalistic design. The five programs all have a similar structure and look. Each program addresses a particular anxiety disorder (ie, *GAD Online* treats GAD, *Panic Stop!* treats PD/A, *OCD Stop!* treats OCD, *PTSD Online* treats PTSD, and *SAD Online* treats SAD). Each program is based on well-established CBT principles and protocols and was reviewed by national and international experts. In addition, all programs were subjected to rigorous technical and consumer usability testing prior to launch.

All programs consist of 12 modules, delivered over 12 weeks, that include a variety of text-based and multimedia materials (audio, video, and animated graphics) and online activities—for example, video (expert speaking, patient speaking, examples of therapy techniques or sessions, etc), audio (breathing control, visual imagery, progressive relaxation therapy instructions, etc), online activities (weekly self-monitoring, quizzes, journal writing, etc), downloadable PDFs (worksheets, transcripts of the audio, monitoring forms, etc), and online interactive animations (flash animations to convey key concepts) (see Multimedia Appendix 1 for several screenshot examples). In addition, there were numerous automated emails welcoming participants to the program, reminding and encouraging them to log on and complete their assessments, as well as various “alert” emails that are triggered depending on participants’ online behavior (eg, alert automated emails are triggered when participants’ self-monitoring of their anxiety and depression remain static for 4 weeks, or remains in the upper extreme range for 2 weeks in a row). These automated emails alert the person to a particular issue and provide recommendations (eg, to consider seeking more intensive assistance). All e-therapy programs contain standard CBT content teachings with regard to psychoeducation, anxiety management, and physiological, cognitive, and behavioral change strategies specific to each anxiety disorder, as well as weekly online and offline homework activities.

### Procedures

Participants self-register to use the Anxiety Online virtual clinic. All participants are required to read and agree to the terms and conditions of the Anxiety Online service before being able to proceed to e-PASS. If participants meet criteria (18 years of age and over, and receiving a diagnosis of GAD, PD/A, OCD, PTSD, or SAD), they are offered the e-therapy program(s) that treat their specific disorder(s).

For this study, once participants chose a fully automated self-help e-therapy treatment program, they gained immediate access to the program and their 12-week e-therapy treatment cycle commenced. During this 12-week period, participants could not undertake another e-therapy program that they may have been offered in their e-PASS report (participants are unable to do more than one e-therapy program concurrently). If participants no longer wanted to continue, they were required to opt out of their e-therapy program via an opt-out option provided within each e-therapy program.

After completing the e-therapy program (at the end of week 12), participants who had not opted out were sent automated emails asking them to complete their posttreatment assessment questions and e-PASS. Several reminder emails were sent out over a 3-week period to those who had not completed the posttreatment assessment in a timely manner.

#### Statistical Procedures, Analyses, and Evaluation of Treatment Effects

After multivariate analysis of variance (MANOVA) tests showed no significant attrition bias, treatment effects from pre- to postassessment were evaluated separately for each of the five fully automated self-help e-therapy treatment programs, using a repeated measures MANOVA for the five treatment outcome variables (ie, clinical disorder severity rating, K6 scores, number of diagnoses, confidence in managing one’s own mental health care, and quality of life). Follow-up analysis of variance (ANOVA) tests were then conducted for the programs with significant results. Normality and homogeneity assumptions were supported by the data and effect sizes were established using Cohen’s [44] classification scheme (small effect = 0.20, medium effect = 0.50, and large effect = 0.80). The 95% confidence intervals for the expected program changes are also presented. In addition, the e-therapy treatment satisfaction results are presented. We used SPSS version 19 for Windows (IBM Corporation, Somers, NY, USA) to analyze all data.

#### Attrition

**Figure 1:**
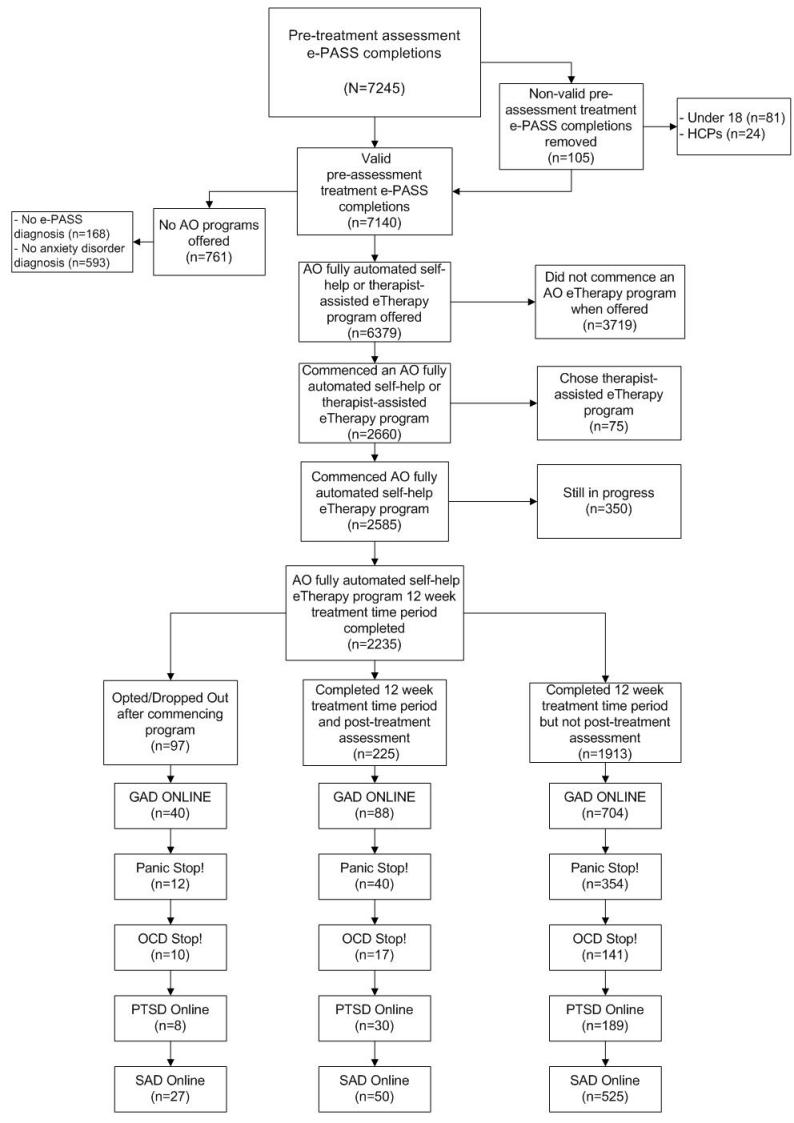
Recruitment flow (AO = Anxiety Online, e-PASS is an online psychological assessment system, GAD = generalized anxiety disorder, HCP = health care professional, OCD = obsessive–compulsive disorder, PTSD = posttraumatic stress disorder, SAD = social anxiety disorder).

The overall completion rate, for the purposes of this study, was defined as the number of participants who started a program and completed the 12-week posttreatment assessment (postassessment completion rate of 10.1% [225/2235], or 89.9% attrition rate). However, true attrition, as defined by those participants who opted out or dropped out of a program after commencement, was much lower at just 4% (97/2235). The bulk of participants (n = 1913) were those who commenced a program, did not opt or drop out, and did not complete their posttreatment assessment. In this situation the most reliable measure of attrition probably ignores this group, producing an attrition rate of 30.1% (225/322 = 69.9% completed). As Figure 1 shows, the attrition rates for the five treatment programs were fairly similar when this attrition measure was used (31% for GAD, 23% for Panic Stop!, 37% for OCD Stop!, 21% for PTSD and, 35% for SAD).

These data clearly illustrate the inherent completion difficulties facing e-mental health evaluation research using open-access research designs. It is therefore necessary to check for attrition bias using robust statistical techniques (eg, [45,46]). In cases where attrition bias is not found to be significant, the use of completer analysis is considered a legitimate and accurate means to analyze the data. However, in cases where significant attrition bias is found, the more conservative intention-to-treat method should be applied. Below we present two methods of checking for attrition bias, although either one is sufficient.

#### Methods for Checking for Attrition Bias

We used two methods to check for attrition bias. The first method was initially proposed by Heckman [45]. In this approach, nominal logistic regression is used to predict the probability that each participant will complete the posttreatment assessment using a variety of pretreatment assessment and demographic measures. The Mills ratio is then produced using the ratio of the normal probability and cumulative distribution function for the residuals (1 minus the predicted probability for posttreatment assessment completion). This Mills ratio is then included as a covariate in a multivariate general linear model to determine the effect of the program on the posttreatment minus pretreatment changes in the outcome measures. If the Mills ratio is not significant, it indicates that the responses for those who did complete the posttreatment assessment are indicative of what could be expected for those who did not complete the posttreatment assessment; in other words, there is no attrition bias.

The second approach has been used by authors such as Rubin [46] to allow for propensity subclassification. In this study, propensity is the estimated probability of attrition developed using the nominal logistic regression procedure. In this study, we used the quartiles for this estimated probability to create subclassifications on which a multivariate general linear model is used to test for main effects and an interaction effect between the programs and the attrition propensity quartile effects. If no significant interaction effect and no significant quartile effect are found, it means that the program effects are similar across quartiles, suggesting that there is no significant attrition bias.

#### Check For Attrition Bias

We found nine pretreatment demographic variables to be significantly associated with attrition. As indicated in Table 1, both types of attrition (e-therapy program completed but no posttreatment assessment, and genuine attrition as defined by formally dropping our or opting out during treatment) were considered in this analysis. The results suggest that those who completed the posttreatment e-PASS tended to differ from the participants who did not in the following ways. It was more likely that on average the completers sought online assistance with the prime objective of finding a self-help program; were married or cohabiting with their partner; were not a homemaker, on a disability pension, or unemployed; were living in a regional area; were more likely to say that they had adequate support; were more likely to say that they learned by reading; had a lower pretreatment K6 score; had a higher age; and had fewer disorders diagnosed at pretreatment assessment. These differences made it necessary for special tests for attrition bias to be performed. These tests showed that none of the attrition-linked variables was associated with changes in the outcome variables, thereby confirming that there was no attrition bias.

**Table 1:** Predictor analysis for attrition categories

Variable		Attrition category						Test of association	
		No attrition: 12-week treatment period completed and posttreatment assessment (n = 225)		Opted out (n = 97)		12-week treatment period completed but not posttreatment assessment (n = 1913)		Test statistic value	*P* value
		%	n	%	n	%	n		
**Reason for seeking online assistance**								χ^2^_2_ = 14.6	.001
	To complete one of the self-help programs	66	149	52	50	53	1014		
**Marital status**								χ^2^_22_ = 32.3	.001
	Married	43	96	40	39	35	668		
	Single	24	55	27	26	29	545		
	Cohabiting	17	39	13	13	19	367		
	Other	16	35	20	19	17	331		
**Employment status**								χ^2^_12_ = 31.6	.002
	Full-time	36	80	31	30	41	788		
	Part-time	25	56	26	25	25	473		
	Home, disability, or unemployed	19	46	24	23	22	411		
	Retired	8	17	4	4	2	42		
	Other	12	26	16	15	10	199		
**Residential setting**								χ^2^_6_ = 14.2	.03
	Metropolitan	62	139	77	75	67	1272		
	Regional	27	61	17	16	22	411		
	Rural	11	25	5	5	11	210		
	Remote	0	0	1	1	1	17		
**Adequate support**								χ^2^_2_ = 8.2	.02
	Yes	50	113	33	32	46	878		
**Preferred learning style**								χ^2^_2_ = 12.3	.06
	Hearing	8	19	6	6	6	113		
	Reading	36	82	31	30	29	545		
	Looking	13	29	14	14	19	363		
	Doing	42	95	49	47	47	891		
		Mean	SD	Mean	SD	Mean	SD		
**Kessler-6**		16.14	5.02	16.86	4.85	17.05	4.84		
**Age (years)**		42.08	12.51	37.96	13.12	36.64	11.94		
**Number of disorders diagnosed**		4.40	2.12	4.78	2.25	4.90	2.22		

We used a nominal logistic regression analysis to predict the attrition category for all participants on the basis of the above nine variables. The estimated probability of completion for the posttreatment assessment was saved for each person who actually completed the posttreatment assessment.

Using the Heckman [45] approach, the Mills ratio for the 225 people who completed the posttreatment assessment was calculated as described above, and a MANOVA was run for the change in all the metric outcome variables to test for differences in the program effects while controlling for the Mills ratio. We found that the Mills ratio had no significant effect (*F*_9,207_ = .686, *P* = .72) and that there was no significant interaction between the program and the Mills ratio (*F*_36,840_ = .854, *P* =.71). This confirms that there is unlikely to be any attrition bias for any of the e-therapy programs.

Next, we used the propensity subclassification approach [46] to split the sample of the 225 participants who completed the posttreatment assessment into four groups. The groups were differentiated in terms of the likelihood of attrition using the quartiles for this estimated probability and were of similar size. Effectively this differentiation controls for the likelihood of attrition, allowing us to determine whether there is attrition bias. A 2-way MANOVA was run for the change in all the outcome variables allowing for an interaction effect between the attrition propensity quartiles and the programs. Neither the interaction effect nor the main effect for the attrition propensity quartiles was found to be significant (*F*_108,1845_ = 1.078, *P* = .28; *F*_27,597_ = .810, *P* = .74), confirming that there is unlikely to be any attrition bias for any of the programs.

In summary, both techniques for assessing attrition bias delivered nonsignificant findings and demonstrate that attrition bias was highly unlikely for all five of the e-therapy programs. Given this result, we analyzed the data for each of the five fully automated e-therapy anxiety disorder treatment programs using a completer analysis.

#### Power Analysis

Target sample size required was determined by GPower [47]. To achieve power of 80% (alpha =.05), 34 participants per e-therapy treatment group were required to detect a moderate effect size on the primary outcome measure (clinical severity rating). Three of the five e-therapy treatment program groups had >34 participants (GAD Online, n = 88; SAD Online, n = 50; Panic Stop!, n = 40); however, PTSD Online and OCD Stop! reached only 30 and 17, respectively, so these results should be interpreted with greater caution.

## Results

### Participant Characteristics

A total of 225 people met the inclusion criteria and completed both pre- and posttreatment assessments. Across the five e-therapy programs, 69 men participated (69/225 = 31%) with the average age of all participants being 42.1 (SD 12.5) years (men, mean 44.5, SD 13.4; women, mean 41.0, SD 12.0 years). The overwhelming majority of participants were Australian residents (215/225 = 95.6%).

Table 2 presents demographic information for each of the five e-therapy program groups.

**Table 2:** Demographic results (n, %) of the posttreatment assessment completers by each one of the five Anxiety Online fully automated self-help e-therapy treatment programs

Demographic variable		GAD^a^ Online (n = 88)		Panic Stop! (n = 40)		OCD^b^ Stop! (n = 17)		PTSD^c^ Online (n = 30)		SAD^d^ Online (n = 50)	
		%	n	%	n	%	n	%	n	%	n
**Gender**											
	Male	33	29	40	16	53	9	13	4	22	11
	Female	67	59	60	24	47	8	87	26	78	39
**Age category (years)**											
	18–24	8	7	5	2	6	1	10	3	10	5
	25–34	18	16	20	8	24	4	23	7	34	17
	35–44	27	24	40	16	41	7	20	6	20	10
	45–54	23	20	20	8	12	2	30	9	20	10
	55–64	21	18	13	5	18	3	13	4	10	5
	65–74	2	2	3	1	0	0	3	1	4	2
	75+	1	1	0	0	0	0	0	0	2	1
**Marital status**											
	Single	19	17	18	7	24	4	37	11	32	16
	Married	51	45	53	21	41	7	23	7	32	16
	Cohabitating	17	15	18	7	24	4	10	3	20	10
	In a relationship but not living together	7	6	10	4	0	0	3	1	8	4
	Separated/divorced and not in a relationship	5	4	0	0	12	2	17	5	6	3
	Widowed and not in a relationship	0	0	3	1	0	0	10	3	2	1
	Other	1	1	0	0	0	0	0	0	0	0
**Australian resident**											
	Yes	97	85	95	38	94	16	93	28	96	48
**Residential setting**											
	Metropolitan	66	58	58	23	71	12	50	15	62	31
	Regional	25	22	30	12	24	4	37	11	24	12
	Rural	9	8	13	5	6	1	13	4	14	7
	Remote	0	0	0	0	0	0	0	0	0	0
**Secondary education**											
	Did not complete primary school	1	1	0	0	0	0	3	1	0	0
	Completed primary school	0	0	0	0	0	0	0	0	0	0
	Completed secondary up to year 9	1	1	3	1	0	0	7	2	4	2
	Completed secondary year 10	10	9	15	6	0	0	13	4	14	7
	Completed secondary year 11	5	4	5	2	0	0	10	3	8	4
	Completed secondary year 12	83	73	78	31	100	17	67	20	74	37
**Highest level of tertiary education**											
	None	8	7	25	10	6	1	17	5	12	6
	Apprenticeship/trade	1	1	8	3	0	0	3	1	4	2
	Other certificate	2	2	3	1	0	0	27	8	12	6
	Diploma	11	10	3	1	6	1	17	5	8	4
	Current undergraduate	11	10	10	4	0	0	3	1	10	5
	Completed undergraduate	36	32	23	9	53	9	13	4	28	14
	Postgraduate	28	25	28	11	35	6	17	5	16	8
	Other	1	1	3	1	0	0	3	1	10	5
**Employment status**											
	Employed full-time	36	32	40	16	47	8	30	9	30	15
	Employed part-time/casual	26	23	35	14	29	5	23	7	14	7
	Home duties	6	5	3	1	0	0	10	3	14	7
	Disability support	0	0	3	1	12	2	3	1	2	1
	Unemployed	7	6	8	3	6	1	17	5	20	10
	Retired	9	8	8	3	6	1	3	1	8	4
	Other	16	14	5	2	0	0	13	4	12	6
**Currently taking an antidepressant or benzodiazepine medication?**											
	Yes	26	23	35	14	23	4	30	9	12	6
**Currently receiving mental health assistance?**											
	Yes	42	37	50	20	41	7	57	17	22	11
**Diagnosed physical health condition?**											
	Yes	40	35	33	13	35	6	47	14	36	18
**Stage of change**											
	Not interested or no need at this time	0	0	3	1	0	0	0	0	0	0
	Neither here nor there	0	0	3	1	0	0	10	3	6	3
	Prepared to take action	57	50	43	17	59	10	53	16	48	24
	Already making changes	39	34	33	13	35	6	30	9	34	17
	Relapsed and looking for additional assistance	5	4	20	8	6	1	7	2	12	6
**Do you feel you have an adequate level of social support or enragement in social/community activities?**											
	Yes	42	37	70	28	65	11	57	17	40	20
**Preferred learning style**											
	Hearing	5	4	15	6	18	3	0	0	12	6
	Reading	40	35	38	15	29	5	33	10	34	17
	Looking/watching	10	9	15	6	12	2	13	4	16	8
	Doing	46	40	33	13	41	7	53	16	38	19

^a^ Generalized anxiety disorder.

^b^ Obsessive–compulsive disorder.

^c^ Posttraumatic stress disorder.

^d^ Social anxiety disorder.

### Treatment Outcomes

The number, means, standard deviations, *F* scores, *P* values, Cohen d, and confidence intervals for the five key dependent variables (per e-therapy program group) at the two assessment periods are shown in Table 3.

**Table 3:** Pre- and posttreatment assessment by Anxiety Online e-therapy treatment program group for posttreatment completers

Variable by e-therapy program disorder type		n	Mean	SD	*F*_1,n – 1_	*P* value	Cohen d (within groups)	95% CI^a^
**GAD^b^ Online**		88						
	GAD CDSR^c^ pre^d^		3.26	1.5				
	GAD CDSR post^e^		1.82	1.6	64.97	<.001	1.22	1.1 to 1.8
	K6^f^ pre		16.64	4.4				
	K6 post		13.65	4.2	58.70	<.001	1.16	2.2 to 3.8
	Disorder number^g^ pre		4.24	1.8				
	Disorder number post		3.17	1.9	34.45	<.001	0.89	0.7 to 1.4
	Confidence^h^ pre		3.12	0.9				
	Confidence post		3.63	0.8	25.18	<.001	0.77	–0.7 to –0.3
	Quality of life^i^ pre		3.37	0.8				
	Quality of life post		3.59	0.8	5.67	.02	0.36	–0.4 to –0.04
**Panic Stop!**		40						
	PD^j^ CDSR pre		3.13	1.9				
	PD CDSR post		1.63	2.2	24.44	<.001	1.12	0.9 to 2.1
	K6 pre		15.18	4.5				
	K6 post		13.43	4.9	12.79	.001	0.81	0.8 to 2.7
	Disorder number pre		4.60	2.2				
	Disorder number post		3.97	2.6	3.92	.055	0.45	–0.01 to 1.3
	Confidence pre		3.03	1.0				
	Confidence post		3.48	0.9	11.32	.002	0.75	–0.7 to –0.2
	Quality of life pre		3.55	1.0				
	Quality of life post		3.60	1.0	0.50	.62	0.11	–0.3 to 0.2
**OCD^k^ Stop!**		17						
	OCD CDSR pre		2.33	0.9				
	OCD CDSR post		1.52	1.8	4.95	.04	0.83	0.04 to 1.6
	K6 pre		14.06	6.2				
	K6 post		13.47	6.6	0.45	.51	0.23	–1.3 to 2.5
	Disorder number pre		3.29	1.4				
	Disorder number post		2.12	1.3	9.79	.006	1.08	0.4 to 2.0
	Confidence pre		3.18	0.8				
	Confidence post		3.76	0.9	11.59	.004	1.17	–1.0 to –0.2
	Quality of life pre		3.71	1.1				
	Quality of life post		4.00	1.1	6.67	.02	0.87	–0.5 to –0.1
**PTSD^l^ Online**		30						
	PTSD CDSR pre		3.17	1.6				
	PTSD CDSR post		1.98	1.8	6.71	.02	0.72	0.3 to 2.1
	K6 pre		18.53	5.2				
	K6 post		14.20	5.7	13.54	.001	0.95	1.9 to 6.7
	Disorder number pre		5.33	2.6				
	Disorder number post		4.00	2.9	10.55	.003	0.85	0.5 to 2.2
	Confidence pre		3.03	1.0				
	Confidence post		3.83	0.9	18.08	<.001	1.08	–1.2 to –0.4
	Quality of life pre		2.97	0.9				
	Quality of life post		3.50	0.9	14.17	.001	0.96	–0.8 to –0.2
**SAD^m^ Online**		50						
	SAD CDSR pre		3.10	1.7				
	SAD CDSR post		2.20	2.0	16.73	<.001	0.84	0.4 to 1.3
	K6 pre		15.30	5.3				
	K6 post		14.26	4.9	2.42	.13	0.31	–.3 to 2.4
	Disorder number pre		4.32	2.3				
	Disorder number post		3.74	2.3	6.33	.02	0.50	0.1 to 1.0
	Confidence pre		2.90	1.0				
	Confidence post		3.44	0.9	13.13	.001	0.70	–0.8 to –0.2
	Quality of life pre		3.24	0.9				
	Quality of life post		3.52	0.9	6.84	.01	0.51	–0.5 to –0.1

^a^ Confidence interval (mean difference).

^b^ Generalized anxiety disorder.

^c^ e-PASS (online psychological assessment and referral system) clinical disorder severity rating, range 0–8.

^d^ pre = preassessment.

^e^ post = postassessment.

^f^ Kessler6, range 6–30.

^g^ Disorder number = number of disorders assessed by e-PASS, range 0–21.

^i^ Quality-of-life ratings range 1–5.

^j^ Panic disorder.

^k^ Obsessive–compulsive disorder.

^l^ Posttraumatic stress disorder.

^m^ Social anxiety disorder.

#### GAD Online Program

For the GAD Online program a repeated measures MANOVA revealed a significant multivariate time effect for the e-PASS severity rating, K6, e-PASS total, quality-of-life, and confidence outcome measures (*F*_5,83_ = 19.92, *P* < .001). Follow-up repeated measures ANOVAs revealed significant improvements on all five variables. Three of the five treatment outcome variables produced large effect sizes, with one medium effect size and one small effect size (see Table 3).

#### Panic Stop! Program

For the Panic Stop! program a repeated measures MANOVA with these variables revealed a significant multivariate time effect (*F*_5,35_ = 8.87, *P* < .001). Follow-up repeated measures ANOVAs on three of the five variables revealed significant improvements on three variables. Two of these variables produced large effect sizes, with one medium effect size, one small effect size, and one very small (under .20) (see Table 3).

#### OCD Stop! Program

For the OCD Stop! program a repeated measures MANOVA with these variables revealed a significant multivariate time effect (*F*_5,12_ = 4.21, *P* = .02). Follow-up repeated measures ANOVAs revealed significant improvements on four of the five variables. Four of these variables produced large effect sizes with one small effect size (see Table 3).

#### PTSD Online Program

For the PTSD Online program a repeated measures MANOVA with these variables revealed a significant multivariate time effect (*F*_5,25_ = 4.89, *P* = .003). Follow-up repeated measures ANOVAs revealed significant improvements on all five variables. Four of these outcome variables produced large effect sizes with one medium effect size (see Table 3).

#### SAD Online Program

For the SAD Online program, a repeated measures MANOVA revealed a significant multivariate time effect for these variables (*F*_5,45_ = 5.14, *P* = .001). Follow-up repeated measures ANOVAs revealed significant improvements on four of the five variables; however, only one of these variables produced a large effect size, with three medium effect sizes and one small effect size (see Table 3).

### e-Therapy Program Treatment Satisfaction and Time Spent using the e-Therapy Program

Satisfaction with the e-therapy programs was rated as moderately high on average within all five groups (see Table 4), with the PTSD Online group obtaining the highest average score (3.73/5.00 = 74.6%). In terms of how much the participants liked their e-therapy program, average scores fell into the “somewhat” to “quite a lot” range, with the PTSD Online group likeability score the highest (5.67/8.00 = 70.9%). Chi-square tests indicated no significant differences between the five e-therapy program groups on the two e-therapy program satisfaction questions (χ^2^_16_ = 12.8, *P* = .69; χ^2^_16_ = 16.3, *P* = .43). Posttreatment assessment completers were also asked how much time they spent using their respective e-therapy programs over the 12 weeks. GAD Online participants reported the most amount of time and the OCD Stop! participants the least amount of time (see Table 4). An ANOVA indicated no significant differences between the five e-therapy program groups with respect to the amount of time in minutes spent using their program over the 12-week treatment period (*F*_4,220_ = 0.176, *P* = .95). The average total time in minutes across the five different e-therapy programs was 395.60 (SD 277.2) minutes or 6.59 hours over 12 weeks.

**Table 4:** e-Therapy program treatment satisfaction and likability ratings and time spent using their e-therapy program over the 12-week treatment period

Satisfaction and program usage variable	GAD^a^ Online (n = 88)		Panic Stop! (n = 40)		OCD^b^ Stop! (n = 17)		PTSD^c^ Online (n = 30)		SAD^d^ Online (n = 50)	
	Mean	SD	Mean	SD	Mean	SD	Mean	SD	Mean	SD
How satisfied are you with the online treatment program that you undertook?^e^	3.53	1.0	3.68	0.9	3.65	0.9	3.73	0.8	3.42	0.9
How much did you like the online program?^f^	5.23	2.3	5.45	1.8	5.41	1.8	5.67	1.6	5.20	1.8
How many hours did you spend in total reading/viewing the content in the online program? (minutes)	402.61	283.8	390.75	249.3	344.12	224.0	402.00	268.5	400.80	293.3

^a^ Generalized anxiety disorder.

^b^ Obsessive–compulsive disorder.

^c^ Posttraumatic stress disorder.

^d^ Social anxiety disorder.

^e^ Rating scale: 1 = not at all, 2 = slightly, 3 = moderately, 4 = highly, 5 = very highly.

^f^ Rating scale: 0 = not at all, 2 = a little, 4 = somewhat, 6 = quite a lot, 8 = very much so.

### Reasons for Opting Out

Participants who opted out of the Anxiety Online program (n = 97) were asked to check one or more items regarding what barrier(s) prevented them from completing their Anxiety Online program when they opted out. Table 5 presents the item(s) endorsed.

**Table 5:** Endorsed barriers that prevented those who opted out from completing their Anxiety Online program (n = 97)

Barriers preventing program completion	%	n
None—got what I needed	29	28
Time pressures	21	20
Lack of motivation	14	14
Too anxious about the content	11	11
Became able to access face-to-face assistance	8	8
Realized I preferred face-to-face assistance	7	7
Internet connection or computer problems	5	5
The program did not seem very useful	5	5
Found the program boring	3	3
Found the program unhelpful	3	3
The program was too hard to navigate	3	3
The program material was too hard to understand	3	3
The program was going to take too long to do	2	2
There was too much text to read	2	2
Too anxious using the computer	1	1
The screen was hard to read (text was too small*)*	0	0

### Basic Professional Labor Time Cost Analysis

The Australian Psychological Society [48] schedule of recommended fees for psychological services assumes the following costs: consultation session 45–60 minutes = AU $212; clinical assessment session 76–90 minutes = AU $308. If one were to use these fees to calculate the professional human labor time costs associated with all the e-PASS pretreatment assessments undertaken (n = 7140) and uptake of all the Anxiety Online e-therapy treatment programs since launch (n = 2563; 2660 – 97 opt-outs), the human labor time costing would equate to AU $8.7 million. The cost of developing Anxiety Online and ongoing maintenance over this period has been close to AU $2.0 million, with the bulk of this amount being a one-off development cost (AU $1.66 million). The labor time cost saving resulting from the Anxiety Online service in the first 18 months of operation is therefore estimated at AU $6.7 million, and into the future the cost savings should be greater, given that the start-up costs will not be recurring expenses.

## Discussion

We observed significant reductions in the GAD, PD/A, OCD, PTSD, and SAD e-PASS diagnostic severity ratings specific to each e-therapy program group and increased self-confidence ratings in managing ones’ own mental health care for all five e-therapy program groups. Cohen d within-group treatment effect sizes were in the high-medium to large categories. Quality-of-life ratings significantly increased for four of the five e-therapy program groupings, with Panic Stop! participants showing little improvement based on the mean scores. Total number of diagnoses was significantly reduced for four of the five programs, and the K6 scores significantly reduced for three of the five e-therapy programs. When looking at the treatment effect sizes for the five program groups over the five measures, 14 were large, six were medium, four were small, and one was under 0.20. These results compare very favorably with other self-help e-therapy treatment programs, which typically have effect sizes between 0.40 and 0.70 [15,31].

Overall, e-therapy treatment satisfaction ratings were good. The total average across all five e-therapy treatment program groups was 72% (3.60/5) for satisfaction and likeability was 67% (5.39/8). Interestingly, these percentages are comparable with those we see for our e-therapy programs provided with therapist assistance [8,14,16,49]. Taking treatment outcome and satisfaction results into account, PTSD Online and GAD Online appear to be the strongest performers of the five e-therapy programs. The total time spent using the fully automated e-therapy programs was under 7 hours over the 12-week treatment period (or just under 33 minutes per week on average).

For those participants who opted out, the main barriers endorsed were none (got what they needed; 29%), time pressures (21%), lack of motivation (14%), and feeling anxious about the content (11%). The least endorsed barriers related to computer anxiety (1%); too much text to read (2%); that the program would take too long to complete (2%); that the material was too hard to understand (3%); that the program was hard to navigate around (3%), unhelpful (3%), boring (3%), or perceived as being not useful (5%);, Internet or computer problems (5%), or preferring (7%) or being able to access to face-to-face therapy (8%). These figures generally support the idea that the Anxiety Online programs themselves are not harmful or detrimental to those participants who opted out. Rather, close to a third of those who opted out prematurely endorsed that they “got what they needed” before the 12-week treatment completion time, and just over a third endorsed time pressures and lack of motivation as reasons for noncompletion.

Although very crude, the basic cost comparison between the development and maintenance costs of Anxiety Online to date relative to the cost of professional human labor using traditional delivery models suggests a 77% saving of AU $6.7 million. However, Anxiety Online and other e-mental health platforms could easily sustain a 10-fold increase in usage without substantially affecting maintenance costs; therefore, the cost savings could be far higher than this into the future. When considered in light of other advantages of e-therapy such as increased consumer access to care due to the removal of traditional barriers such as time, cost, and geographic location restraints, the online-delivery model seems likely to play an increasingly prominent role in modern mental health systems.

### Implications

The major implications of being able to effectively deliver psychological treatment via the Internet relate largely to accessibility. The availability of e-mental health services means that anyone in any location with Internet access can access treatment immediately and at the times and intensities they choose (ie, not limited to scheduled appointments). It also means greater access for those with no or limited access to treatment programs or mental health specialists (eg, living in rural areas, incarcerated), or those who move residences frequently (eg, itinerant workers, armed forces personnel) or have limited mobility (eg, with chronic physical illness, older, disabled). e-Mental health interventions may also help those with mental health problems who are reticent to present to services for reasons such as perceived stigma [50], although recent research supports that some stigmatizing attitudes actually lead to increased likelihood to seek professional help [51].

### Limitations

This study has some limitations that should be taken into consideration. This study was conducted as an open-access, participant choice, naturalistic trial, so the lack of a control group makes it impossible to conclude whether the improvements are a result of the active e-therapy programs or merely a result of other effects. Nevertheless, this design limitation does represent “real-world” mental health services, as consumers are allowed to choose their treatment program rather than being randomly assigned to a treatment program in a randomized controlled trial. Power analyses indicted that the numbers required for the OCD Stop! and PTSD Online e-therapy programs were suboptimal and so some extra caution in interpretation needs to be taken. In previous studies we have used intention-to-treat analysis to address the issue of missing data. Intention-to-treat analysis is an overly conservative approach [52] that potentially underestimates the effectiveness of open-access e-therapy programs, whereas completer analysis may overestimate the effectiveness of these programs if there is attrition bias. Attrition bias was found to be nonsignificant for these data, so there are strong grounds for accepting that the completer analysis results obtained accurately reflect the true effectiveness of the Anxiety Online fully automated self-help programs.

Finally, four of the five dependent variables used in this study lack strong psychometric validation, and this needs to be taken into account when interpreting the results. Further replication studies are required that involve the use of several validated measures before we can definitively confirm that the Anxiety Online fully automated self-help e-therapy programs are effective. Future analyses with the individual Anxiety Online programs will be able to validate the present results with ideal standardized measures, as well as the aforementioned current formal e-PASS psychometric evaluation.

### Future Directions

While results of this open trial are encouraging, it is important that they be followed by randomized controlled trials comparing all five e-therapy programs with both a waitlist control and current best-practice face-to-face treatment in order to unequivocally establish treatment effectiveness. We are running a randomized controlled trial of the OCD Stop! e-therapy program and will be subjecting the other four Anxiety Online e-therapy programs to the same rigorous testing. Furthermore, while e-therapy experts are developing guidelines for working with specific clinical populations (see [53]) as well as Internet intervention research guidelines [54], we also need more research to address the truly important questions about who this modality works best for because, like all forms of treatment delivery, it is unlikely to be universally appealing or effective. We are preparing another paper discussing our preliminary investigations regarding what variables predict attrition for the fully automated self-help e-therapy treatment programs. In addition, we will also be conducting qualitative studies that will include interviewing a random selection of those participants who commenced one of our treatment programs, did not opt out, and did not complete their postassessment. At this point it would be highly speculative to state why so many participants did not complete their postassessment after selecting one of the Anxiety Online programs, apart from pointing to the fully automated nature of the Anxiety Online system (ie, the complete absence of human-based screening, assessment, and therapeutic assistance) to prompt, encourage, and at times enforce adherence behaviors to the treatment protocol.

The Anxiety Online platform will soon be subsumed under the name Mental Health Online, given the addition of new e-therapy treatment programs for nonanxiety conditions (eg, depression, bulimia, insomnia, multidisorder) and several more to follow over the coming months and years (eg, problem gambling, drugs and alcohol, hoarding). We are also integrating other communication modalities into the therapist-assisted programs (ie, instant messaging, audio-only chat, video chat) and the use of 3-dimensional virtual reality platforms and collaborative work spaces. We will offer these new modes of communication and e-therapy training in 2012 and also plan to open up the Anxiety Online/Mental Health Online infrastructure to practitioners in Australia and, potentially, around the world. In the future it will also be possible to access the Anxiety Online/Mental Health Online programs through a national e-mental health portal instigated by the Australian Government Department of Health and Ageing [55]. This portal will bring together many of the evidence-based, yet fragmented, e-mental health interventions currently operating in Australia, thus making it far easier for mental health consumers to find and receive the most appropriate course of treatment with the associated level of assistance that is best for them.

### Conclusions

The results of this open-access participant choice evaluation trial suggest that the Anxiety Online e-therapy programs are promising and effective treatments for people with subclinical and clinical diagnoses of GAD, PD/A, OCD, PTSD, and SAD. e-Mental health treatment-delivery formats are increasing accessibility to mental health care and appear to provide a highly cost-effective and sustainable treatment-delivery model. It is envisaged that e-mental health treatment programs will soon become a common feature of modern mental health systems, and that such a development will bring with it unprecedented levels of service provision to those in need of specialist mental health treatment.

## Multimedia Appendix 1

Four Anxiety Online e-therapy program screenshots providing an example of the use of audio, animations, online activities, and video.

## Abbreviations

ANOVA: analysis of variance
CBT: cognitive behavior therapy
DSM-IV-TR: Diagnostic and Statistical Manual of Mental Disorders: DSM-IV-TR. 4th edition, text revision
e-PASS: online psychological assessment system
