# Supplementary material for: Anxiety Online—A Virtual Clinic: Preliminary Outcomes Following Completion of Five Fully Automated Treatment Programs for Anxiety Disorders and Symptoms
Source: J Med Internet Res. 2011 Nov 4;13(4):e89. doi: 10.2196/jmir.1918 (PMC3222205; doi:10.2196/jmir.1918)

Audio example: Progressive Muscle Relaxation exercise in GAD Online

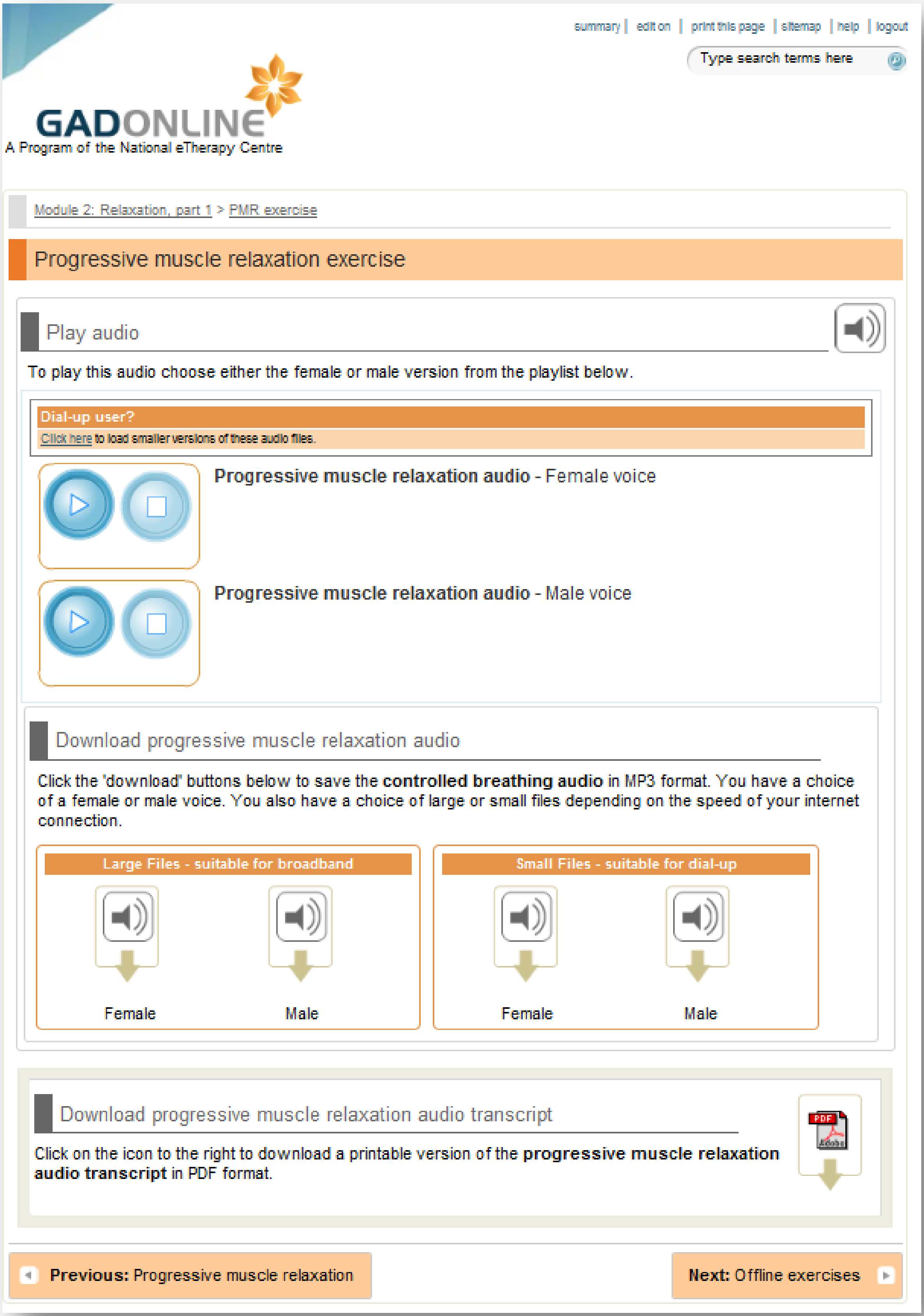

Animation example: Depth of breathing in PTSD Online

PTSDONLINE

A Program of the National eTherapy Centre

Breathing depth check

Depth of breathing

Not only do we want to know about your rate of breathing, but also your **depth** of breathing. Often when anxious we get into the habit of taking shallow breaths, or even holding our breath, which can disrupt your physiology and make you feel on edge. So your breathing rate may be in the normal range (8-10 breaths per minute) but you may be taking shallow breaths.

The diagram below illustrates the differences between shallow and deep breathing.

Examples of Shallow and Deep Breathing

INSTRUCTIONS

Click the start button above the body on the left to see an example of shallow breathing. Click the start button above the body on the right to see an example of deep breathing.

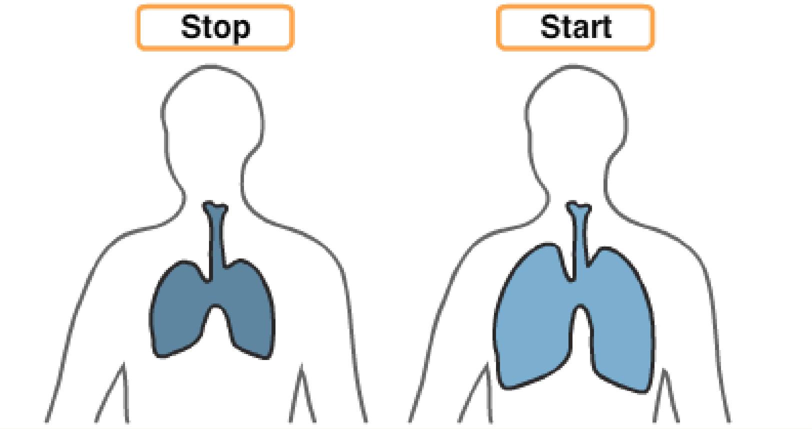

The following simple exercise can tell you more about whether you do shallow or deep breathing.

Exercise

Steps	Action
Step 1	Place one hand on your chest
Step 2	Place one hand on your belly
Step 3	Pay attention to your breathing (it may be easier if you close your eyes)
Step 4	Notice if either or both of your hands move with each breath?

Result

For optimal breathing you should be able to notice the hand on your belly moving, indicating that you are breathing from the belly (diaphragmatic or deep breathing).

Previous: Rate check

Next: Calm breathing

Online Activity example: Problem Solving in OCD Stop!

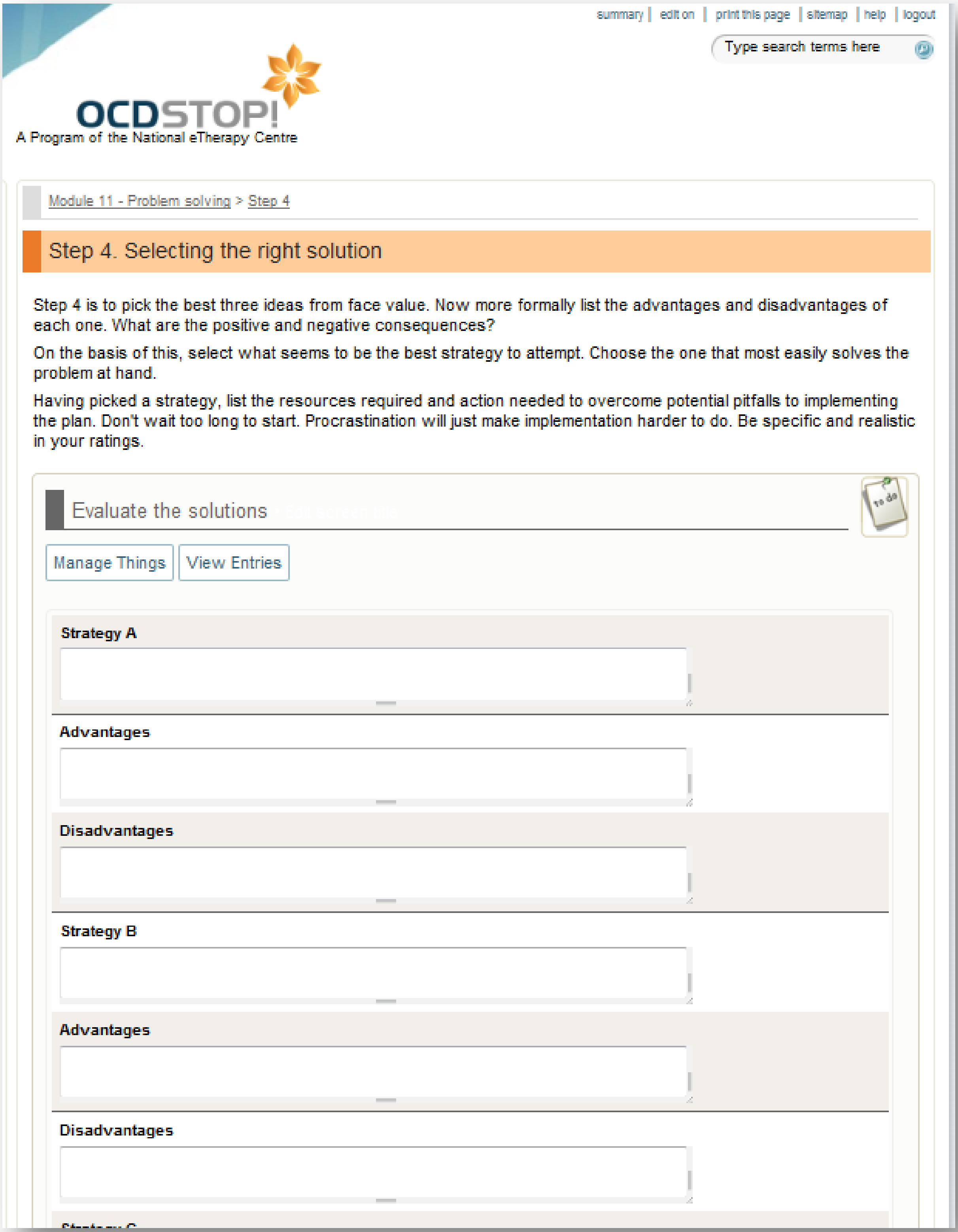

summary | edit on | print this page | sitemap | help | logout

Type search terms here

OCDSTOP!

A Program of the National eTherapy Centre

Module 11 - Problem solving > Step 4

Step 4. Selecting the right solution

Step 4 is to pick the best three ideas from face value. Now more formally list the advantages and disadvantages of each one. What are the positive and negative consequences?

On the basis of this, select what seems to be the best strategy to attempt. Choose the one that most easily solves the problem at hand.

Having picked a strategy, list the resources required and action needed to overcome potential pitfalls to implementing the plan. Don't wait too long to start. Procrastination will just make implementation harder to do. Be specific and realistic in your ratings.

Evaluate the solutions

Manage Things | View Entries

Strategy A

Advantages

Disadvantages

Strategy B

Advantages

Disadvantages

Video example: Exposure exercise in Panic Stop!

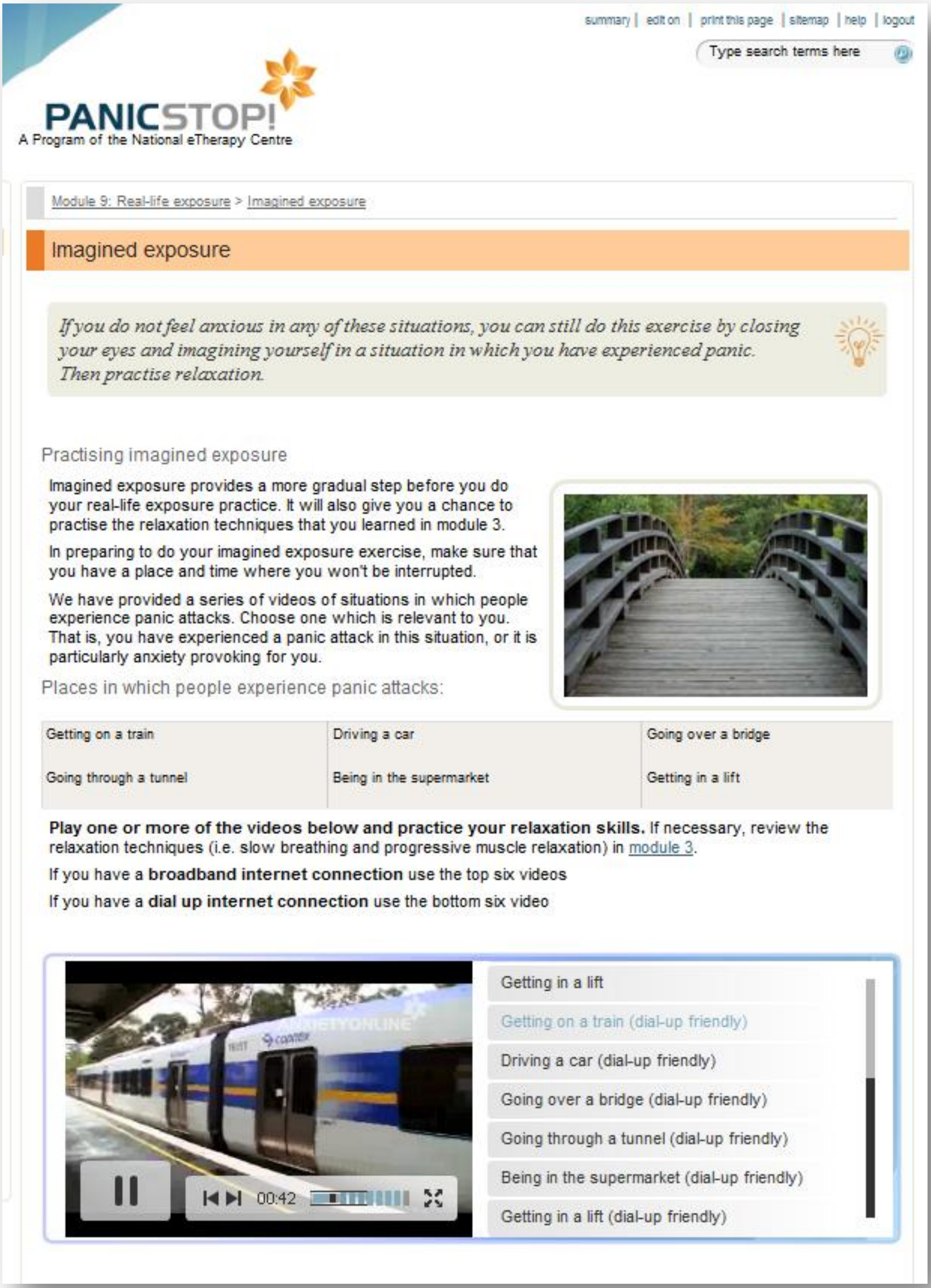

Supplement: Supplementary file 1 [file jmir_v13i4e89_app1.pdf]
